# Prolotherapy injections and physiotherapy used singly and in combination for lateral epicondylalgia: a single-blinded randomised clinical trial

**DOI:** 10.1186/s12891-019-2905-5

**Published:** 2019-11-03

**Authors:** Michael Yelland, David Rabago, Michael Ryan, Shu-Kay Ng, Dinusha Vithanachchi, Nagarajan Manickaraj, Leanne Bisset

**Affiliations:** 1 Menzies Health Institute Queensland, Griffith University, Gold Coast, Queensland 4222 Australia; 2 School of Medicine and Public Health, University of Wisconsin, Madison, USA; 3 School of Allied Health Sciences, Griffith University, Gold Coast, Queensland Australia; 4 Simon Fraser University, Burnaby, British Columbia Canada; 5 School of Medicine, Griffith University, Gold Coast, Queensland Australia

**Keywords:** Elbow, Tendinopathy, Physiotherapy, Prolotherapy, Tennis elbow, Injection, Lateral epicondylalgia

## Abstract

**Background:**

Lateral epicondylalgia (tennis elbow) is a common, debilitating and often treatment-resistant condition. Two treatments thought to address the pathology of lateral epicondylalgia are hypertonic glucose plus lignocaine injections (prolotherapy) and a physiotherapist guided manual therapy/exercise program (physiotherapy). This trial aimed to compare the short- and long-term clinical effectiveness, cost effectiveness, and safety of prolotherapy used singly and in combination with physiotherapy.

**Methods:**

Using a single-blinded randomised clinical trial design, 120 participants with lateral epicondylalgia of at least 6 weeks’ duration were randomly assigned to prolotherapy (4 sessions, monthly intervals), physiotherapy (weekly for 4 sessions) or combined (prolotherapy+physiotherapy). The Patient-Rated Tennis Elbow Evaluation (PRTEE) and participant global impression of change scores were assessed by blinded evaluators at baseline, 6, 12, 26 and 52 weeks. Success rate was defined as the percentage of participants indicating elbow condition was either ‘much improved’ or ‘completely recovered.’ Analysis was by intention-to-treat.

**Results:**

Eighty-eight percent completed the 12-month assessment. At 52 weeks, there were substantial, significant improvements compared with baseline status for all outcomes and groups, but no significant differences between groups. The physiotherapy group exhibited greater reductions in PRTEE at 12 weeks than the prolotherapy group (*p* = 0.014).

**Conclusion:**

There were no significant differences amongst the Physiotherapy, Prolotherapy and Combined groups in PRTEE and global impression of change measures over the course of the 12-month trial.

**Trial registration:**

ACTRN12612000993897.

## Introduction

Lateral epicondylalgia (LE; tennis elbow) is a common, debilitating and expensive musculoskeletal pain condition primarily of the extensor carpi radialis brevis at the lateral humeral epicondyle [1].

While it is generally considered to be self-limiting at six to 24 months [2], up to 10% of patients develop chronic symptoms that are recalcitrant to conservative management and undergo surgical intervention [3, 4].

A physiotherapy program consisting of manual therapy techniques and therapeutic exercise has previously demonstrated clinical effectiveness in the short- and long-term compared to wait-and-see (or placebo injection) and corticosteroid injection [5, 6].

Prolotherapy is an injection therapy that uses a hypertonic irritant solution of glucose with lignocaine, which is thought to stimulate healing and strengthening of degenerative tendon tissue by inciting inflammation followed by collagen deposition and remodelling [7, 8]. Preliminary studies have reported that prolotherapy for LE can result in improved quality of life, pain and function, and may modify the disease course at the level of the damaged tendons [7, 8]. While early clinical trial and anecdotal evidence are promising, prolotherapy for LE lacks high-level evidence of effectiveness.

Whilst there is controlled trial evidence that physiotherapy-directed treatment is more effective than usual care [5, 6] and that prolotherapy injections are more effective than placebo injections [8], physiotherapy-directed treatment and prolotherapy injections have never been directly compared in a pragmatic trial. This is a question of considerable interest to clinicians choosing from the available active treatments and for the patients and health funds who pay for them. Furthermore, given that both exercise and prolotherapy injections are hypothesised to effect clinical outcomes through targeting tissue regeneration [9, 10], the combined effect of prolotherapy with physiotherapy may be proportionally larger than each treatment singly applied. The aim of this study was to investigate the short and long term clinical and cost effectiveness of prolotherapy versus physiotherapy, both singly and in combination in people with LE.

## Materials and methods

### Study design

This was a randomised, single-blinded clinical trial with 1-year follow-up, performed in a community setting in Australia. Ethical approval was obtained from the local university Human Research Ethics Committee (PES/11/12/HREC). The trial was prospectively registered with the Australian New Zealand Clinical Trials Registry (ACTRN12612000993897).

### Participants

Volunteers were included if they were aged 18–70 years and had a clinical diagnosis of LE, defined as pain over the lateral humeral epicondyle of at least 6 weeks’ duration provoked by palpation and resisted wrist/middle finger extension or gripping [11]. In addition, participants needed to score at least 20/100 on the Patient Rated Tennis Elbow Evaluation (PRTEE) and be able to understand enough English to complete the outcome questionnaires. Exclusion criteria included any treatment for their elbow pain by a health care practitioner within the preceding 3 months, concomitant neck or other arm pain causing disability or requiring treatment within the last 6 months, clinical evidence of other primary sources of lateral elbow pain, upper limb fractures within the preceding 10 years, elbow surgery, systemic inflammatory disorder or malignancy, any contraindications to the study treatments, unresolved litigation or workers compensation claims, and pregnancy or breastfeeding.

Participants were recruited from September 2012 to June 2014, via referrals from health professionals and through local media, social and web-based advertising. Eligibility was initially assessed via a telephone screen followed by a clinical assessment by an experienced musculoskeletal physiotherapist. Volunteers meeting eligibility criteria gave informed written consent prior to enrolment by the trial administrator.

Participants were randomised to prolotherapy injections, manual therapy/exercise (physiotherapy) or a combination of both (prolotherapy+physiotherapy), using a computer-generated block randomisation schedule (*N* = 6) generated and administered independently by the University Clinical Trials Centre. The trial administrator assigned the participants to their treatments and liaised with treating practitioners. Study personnel involved in participant screening, treatment and assessment were blind to group allocation throughout the full duration of the trial.

### Sample size

An estimated 120 participants (40 per group) were required to detect a clinically important improvement of 13 points from baseline on the Patient Rated Tennis Elbow Evaluation (PRTEE; α = 0.05, β = 0.1) [12] and to detect a 20% difference between groups in the proportion of participants achieving ‘success’ according to the participant Global Impression of Change (GIC; α = 0.05, β = 0.2), assuming a success rate of 43% in the Combined group, and a 23% rate in the Physiotherapy group, and allowing for 10% loss to follow-up [5].

### Treatment

All participants were provided with written educational material on their condition, with advice to use their affected arms but to avoid activities that resulted in increased pain for several minutes or more. They were encouraged to avoid use of non-trial treatments and asked to record them if they were used. In participants with bilateral LE, both elbows were treated, with the more severely affected side being the focus of outcome assessment and analysis to ensure that it met the eligibility criteria and to avoid a potential source of selection bias.

#### Physiotherapy

A standardised treatment protocol was implemented, based on a previously evaluated program that has demonstrated effectiveness [4, 5]. Four, 30-min treatment sessions were provided at weekly intervals by a post-graduate trained musculoskeletal physiotherapist in a private practice setting. An evidence-based, pragmatic multimodal program, comprising education, manual therapy and therapeutic exercise, was used in conjunction with a home exercise program [4, 12]. Specific manual therapy techniques known as Mobilisation-With-Movement (MWM) were applied. In addition, three main groups of exercises were pragmatically prescribed: (a) Sensorimotor retraining of gripping and posture correction were commenced early in the physiotherapy intervention; (b) progressive resistance exercise for the wrist extensors were prescribed based on identified strength deficits; and (c) exercises geared towards general arm strengthening were also prescribed. The physiotherapist prescribed exercises based on the participant’s capabilities at each session to allow for optimal exercise volume and load setting. The overriding rule for all exercise was that pain should not be provoked during or after exercise. The physiotherapist reviewed the prescribed exercises at the commencement of each treatment session, and monitored adherence to the home program by reviewing a self-reported exercise diary completed by the participant each week.

#### Prolotherapy injection

The injection protocol for prolotherapy was based on one developed in the 1950s [13] and later refined [14], and which is commonly taught to practitioners in the USA [15]. It was delivered at either a general practice or university-based health clinic by one of two general medical practitioners, each with more than 15 years’ experience in prolotherapy treatments. At each visit, the elbow was palpated for tenderness at points regarded as sources of pain in lateral epicondylalgia, i.e., over the lateral epicondyle, supracondylar ridge, radial head, lateral collateral and annular ligaments, and the common extensor tendon and musculotendinous junction. Each tender point was injected with 0.5 to 1.0 ml of solution containing 20% glucose and 0.4% lignocaine using a peppering technique with a 25-gauge needle. The total amount of solution injected depended on the number of tender points, but did not exceed 5 ml. Participants were advised to expect a temporary increase in pain for a few days following treatment, and to avoid anti-inflammatory medications during this period as they could theoretically reduce the effect of the injections. Non-prescription analgesics were permitted as required. The injections were repeated at 4, 8 and 12 weeks after the initial treatment session. Participants could exit this protocol early if there was either a full recovery or persistent worsening of elbow pain at any stage.

#### Combined treatment

This involved both protocols described above, but with the physiotherapy protocol timed for 1, 2, 3 and 5 weeks after the first prolotherapy treatment to minimise exacerbation of any post-injection soreness.

### Outcome assessment

Baseline demographic and clinical characteristics included age, sex, body mass index, duration of current condition, affected side, hand dominance, occupation, current work status, income, physical activity status, current medications and smoking status. The primary outcomes assessed in this trial were the PRTEE and the participant’s perceived Global Impression of Change (GIC). The PRTEE is a condition-specific self-reported questionnaire comprising five pain items and 10 functional disability items on 11-point numerical rating scales. The PRTEE has excellent test-retest reliability (r = 0.93) and sensitivity to change [16–18], with a clinically important change of 11/100 or 37% from baseline [12]. The GIC used a 6-point Likert scale ranging from ‘much worse’ to ‘completely recovered’. A dichotomous measure of success was defined as either ‘much improved’ or ‘completely recovered’ [5, 6].

Secondary outcomes included validated measures of (a) pain severity, recorded as ‘the level of pain you currently experience at rest’ and ‘the worst level of pain you have experienced in the past 7 days’, each using a 0 to10-point numerical rating scale (0 = no pain at all, 10 = worst pain imaginable) [19]; (b) quality of life via the EuroQoL EQ-5D-3 L scored using Australian weights [20, 21]; and (c) pain-free grip strength (PFG) [22].

The use of medication and other not-per-protocol treatments related to the elbow pain were recorded at each follow-up assessment. The costs of these additional treatments, including costs for general practitioner, medical specialist, or allied health visits, aids and appliances and medications were estimated at contemporary market rates. These costs represented the costs to both the government and the participant. The cost of the trial treatments was calculated from the Australian Medicare Benefits Schedule rebates [23] and the schedule of fees from the local state workers compensation organisation (website accessed 1st September 2014).

Adverse events potentially related to treatment were recorded at all treatment visits and follow-up assessments. Recurrence of condition was defined as participants who moved from a self-reported ‘success’ on follow-up assessment up to 12 weeks, to a ‘non-success’ at 26 or 52 weeks’ follow-up [5, 6]. Compliance with treatment in all groups was defined as a minimum of 75% attendance at treatment sessions; compliance with the exercise protocol was assessed by a questionnaire at each follow-up assessment and was defined as performing the exercises more than twice weekly for the first 12 weeks. All outcome measures were assessed face-to-face with a blinded assessor at baseline and at 6, 12, 26 and 52 weeks with the exception of the GIC, which was not assessed at baseline.

### Data analyses

Demographic and clinical characteristics at baseline were compared between treatment groups to assess the effectiveness of the randomisation procedure. Analyses of outcome data were performed on an intention-to-treat basis by an experienced statistician who was blind to group allocation, using SPSS version 24 (IBM, Chicago, IL). The longitudinal outcome of the PRTEE was analysed using the Generalised Estimating Equation (GEE), with a first-order autoregressive relationship AR [1] working correlation structure to account for within-participant correlation for repeated measurements, and robust estimator for covariance matrix [24]. The GEE is a widely used method for the analysis of longitudinal data. It considers measurements at multiple time points simultaneously and allows for testing the overall significance of the effects. With the GEE, a normal distribution with an identity link was used for scale variable outcomes, while a binomial distribution with a logit link was used for categorical binary variable outcome of success. The assumption of normality within the GEE framework was checked for scale variable outcomes. The effects of treatment, time, and treatment by time interaction were included in all models. The Wald χ^2^ test was used to assess between-group differences and within-group differences in outcomes over time. The GEE works well with missing data, assuming that they are missing completely at random (MCAR) so data imputation was not needed.

Subgroup analyses were performed assessing baseline demographic characteristics for their influence on treatment effects, and reporting adjusted results if they were found to significantly influence outcomes. Differences in medication use and use of other not-per-protocol treatments between groups were analysed using the Chi-squared test. Protocol and not-per-protocol treatment costs were calculated for each group and an analysis of the incremental cost-effectiveness ratio at the follow-up point of maximal differences in the proportion of responders between groups.

## Results

### Participants

**Fig. 1:**
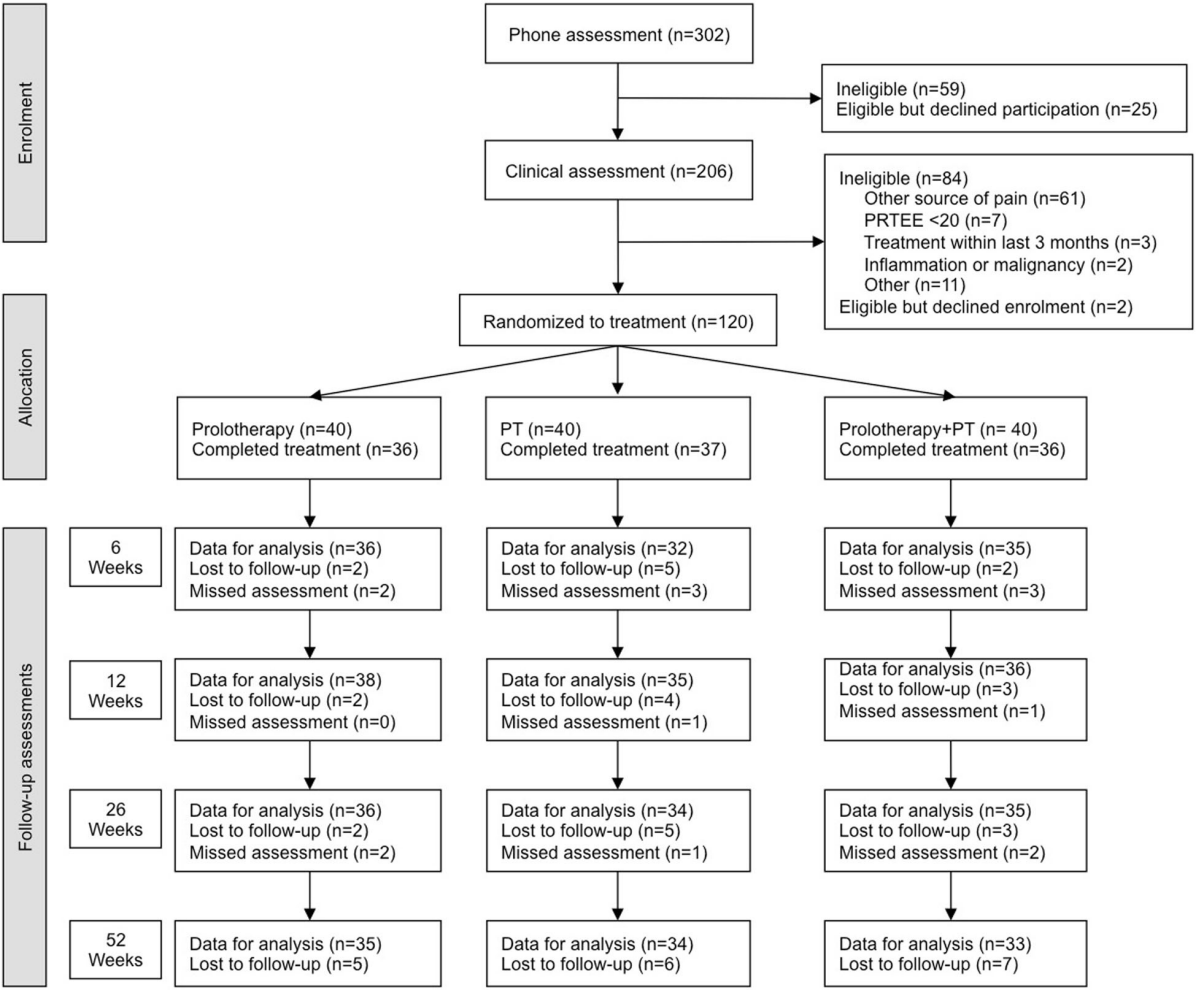
Flow chart of participants through the study

One hundred and twenty participants were included in the analysis (Fig. 1), with > 85% follow-up rates across all groups. The distribution of demographic and clinical characteristics amongst the three groups was similar (Table 1). Compliance rates for treatment was 93% for Prolotherapy, 93% for Physiotherapy and 95% for the Combined group. The outcome assessor correctly guessed group allocation for 31 participants (26%) a guess rate that is less than the random chance rate for three groups (33%).

**Table 1:** Baseline demographic and clinical characteristics of participants by treatment group, reported as mean (standard deviation) unless otherwise specified

Characteristics	Prolotherapy	Combined	Physiotherapy	Total
	*n* = 40	*n* = 40	*n* = 40	*N* = 120
Age, years	49.2 (7.2)	47.8 (7.0)	51.0 (9.0)	49.3 (7.8)
Women, N (%)	18 (45%)	18 (45%)	16 (40%)	52 (43%)
Duration, weeks median, (IQR)	23.0 (22.0)	19.5 (18.0)	21.0 (43.0)	22.0 (27.0)
Employment				
Manual work	17 (42.5%)	20 (50%)	24 (60%)	61 (50.8%)
Non-manual work	19 (47.5%)	13 (32.5%)	11 (27.5%)	43 (35.8%)
Not working	4 (10%)	7 (17.5%)	5 (12.5%)	16 (13.3%)
Previous episode of LE, N (%)				
	12 (30%)	13 (33.3%)	15 (37.5%)	40 (33.6%)
Progress trajectory before study, N (%)				
Better	13 (33.3%)	12 (30.0%)	11 (28.9%)	36 (30.8%)
Worse	13 (33.3%)	17 (42.5%)	12 (31.6%)	42 (35.9%)
Same	13 (33.3%)	11 (27.5%)	15 (39.5%)	39 (33.3%)
Affected side, N (%)				
Right	28 (70%)	19 (47.5%)	24 (60%)	71 (59.2%)
Left	6 (15%)	18 (45.0%)	6 (15%)	30 (25.0%)
Bilateral	6 (15%)	3 (7.5%)	10 (25%)	19 (15.8%)
Dominant side affected, Proportion (%)				
Dominance = Right	32/36 (88.9%)	22/38 (57.9%)	32/36 (88.9%)	86/110 (78.2%)
Dominance = Left	3/4 (75.0%)	2/2 (100.0%)	3/4 (75.0%)	8/10 (80.0%)
PRTEE, /100	31.6 (10.3)	31.3 (10.8)	33.5 (10.0)	32.1 (10.3)
Pain at rest, /10	2.0 (1.6)	1.8 (1.6)	2.1 (2.0)	1.9 (1.7)
Worst pain, /10	7.4 (1.6)	6.1 (2.4)	7.3 (2.0)	6.9 (2.1)
PFG, ratio affected/unaffected	0.56 (0.34)	0.55 (0.32)	0.64 (0.55)	0.58 (0.41)
EuroQoL, /100	82.6 (12.9)	83.1 (11.2)	80.4 (16.9)	82.1 (13.7)

PRTEE = Patient-Rated Tennis Elbow Evaluation; LE = lateral epicondylalgia; PFG = pain-free grip; PPT = pressure pain threshold. ^†^ Between-group comparisons based on chi-square tests for categorical variables and ANOVA for continuous variables (Mantel-Haenszel test for homogeneity of odds ratios for dominant side affected)

### Treatment

**Fig. 2:**
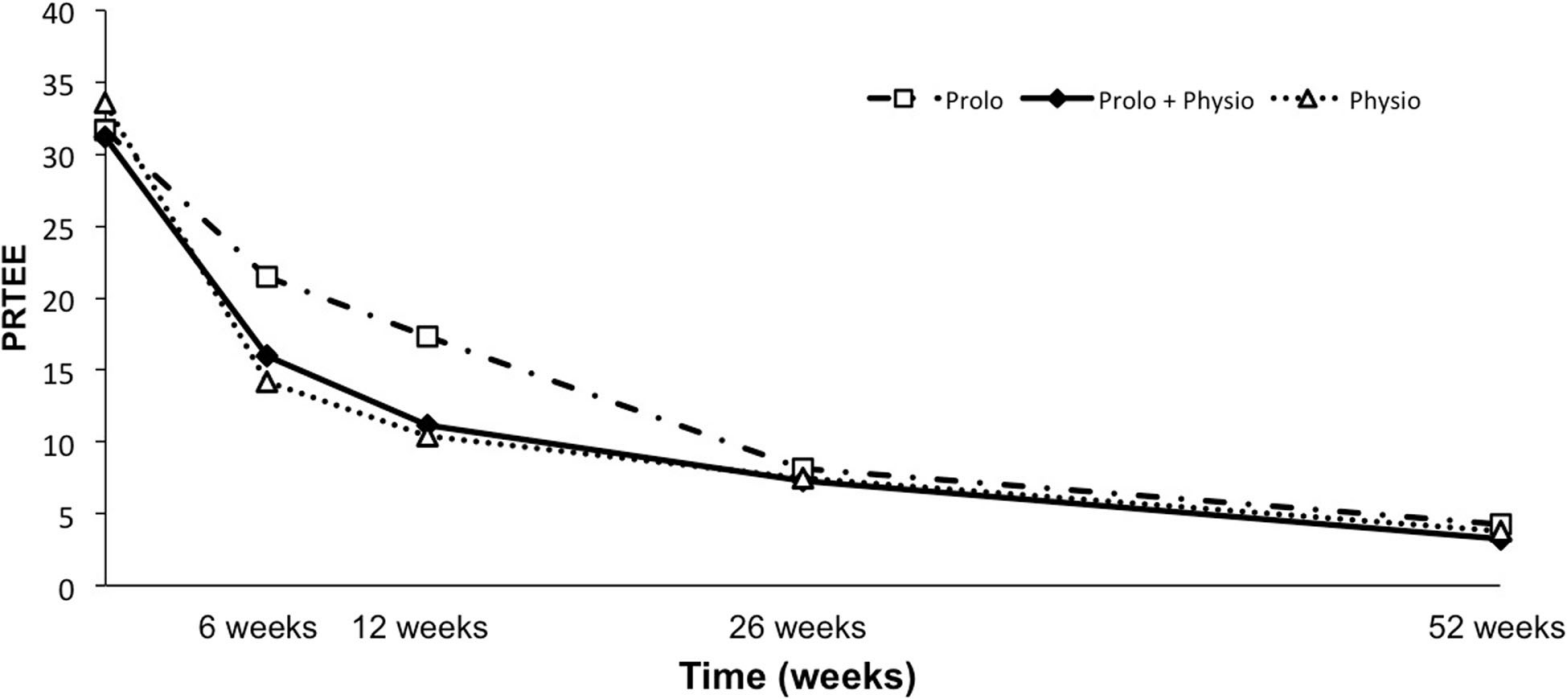
PRTEE scores by group over time

Overall, there was no significant difference in PRTEE between groups over time in the omnibus analysis (*p* = 0.23; Table 2). Similarly, there was no significant difference between groups in terms of the proportion of those achieving the minimum clinically important reduction of 37% in PRTEE scores from baseline (*p* = 0.77). However, all groups demonstrated a significant improvement in PRTEE over time (*p* < 0.001). In the short term, reduction in PRTEE scores was significantly greater at 12 weeks (*p* = 0.01) for Physiotherapy compared to Prolotherapy (Table 2; Fig. 2). Most participants reported a successful outcome at 26 and 52 weeks’ follow up, but there were no significant differences between groups at either time point (Table 2).

**Table 2:** Effects of Prolotherapy, Physiotherapy, and Combined treatments on primary and secondary outcomes at all time points over 1 year follow-up

Outcome	Prolotherapy	Combined	Physiotherapy	Combined vs Prolotherapy	Physiotherapy vs Prolotherapy	Physiotherapy vs Combined
PRTEE, /100 Mean (SD)				Mean improvement from baseline (95% CI)^†^		
0 weeks	31.6 (10.3)	31.3 (10.8)	33.5 (10.0)	–	–	–
6 weeks	24.5 (14.6)	18.3 (12.2)	19.7 (14.3)	5.35 (−1.77, 12.5)	6.31 (−0.83, 13.5)	0.96 (− 6.23, 8.16)
12 weeks	18.2 (13.5)	12.4 (10.1)	12.2 (12.4)	5.21 (− 0.99, 11.4)	7.42 (1.51, 13.3)*	2.21 (−4.29, 8.70)
26 weeks	8.9 (8.2)	8.2 (10.5)	9.3 (10.4)	−0.11 (−6.21, 5.99)	1.01 (−4.56, 6.58)	1.12 (−4.99, 7.24)
52 weeks	4.9 (7.4)	3.9 (5.5)	4.4 (7.0)	0.35 (−4.91, 5.61)	2.10 (−3.31, 7.51)	1.75 (−2.94, 6.45)
Pain at rest, /10						
0 weeks	2.0 (1.6)	1.8 (1.5)	2.1 (2.0)	–	–	–
6 weeks	1.9 (2.0)	1.3 (1.9)	1.5 (1.5)	0.4 (−0.7, 1.4)	0.7 (− 0.2, 1.7)	0.4 (− 0.6, 1.3)
12 weeks	0.8 (1.3)	0.8 (1.2)	1.0 (1.5)	−0.3 (−1.04, 0.5)	0.0 (− 0.8, 0.8)	0.3 (− 0.5, 1.0)
26 weeks	0.3 (0.7)	0.5 (1.7)	0.8 (1.3)	−0.4 (−1.2, 0.5)	− 0.3 (− 1.2, 0.5)	0.1 (− 0.9, 1.0)
52 weeks	0.2 (0.5)	0.2 (0.5)	0.2 (0.6)	− 0.2 (− 0.9, 0.5)	0.1 (− 0.6, 0.9)	0.3 (− 0.4, 1.1)
Worst pain in the last week, /10						
0 weeks	7.4 (1.6)	6.1 (2.4)	7.3 (2.0)	–	–	–
6 weeks	5.4 (2.2)	3.7 (2.3)	3.7 (2.6)	0.2 (−0.9, 1.4)	1.5 (0.5, 2.6)*	1.3 (0.1, 2.5)*
12 weeks	4.0 (2.5)	3.0 (2.1)	2.5 (2.6)	−0.4 (−1.6, 0.8)	1.4 (0.2, 2.6)*	1.7 (0.6, 2.9)*
26 weeks	2.0 (2.0)	2.1 (2.1)	1.6 (2.1)	−1.5 (−2.7, −0.2)*	0.2 (− 1.1, 1.5)	1.7 (0.3, 3.0)*
52 weeks	1.1 (2.0)	0.9 (1.6)	0.9 (1.6)	−1.1 (−2.3, 0.1)	0.0 (− 1.0, 1.1)	1.1 (0.0, 2.2)*
PFG, affected/unaffected ratio						
0 weeks	0.56 (0.34)	0.55 (0.32)	0.64 (0.55)	–	–	–
6 weeks	0.87 (0.57)	0.84 (0.66)	0.80 (0.34)	−0.03 (−0.30, 0.24)	−0.11 (− 0.37, 0.15)	−0.08 (− 0.31, 0.15)
12 weeks	0.79 (0.31)	0.81 (0.46)	1.00 (0.50)	0.02 (−0.16, 0.19)	0.12 (−0.13, 0.37)	0.10 (− 0.14, 0.34)
26 weeks	0.92 (0.23)	0.89 (0.39)	1.03 (0.35)	−0.03 (− 0.22, 0.17)	0.03 (− 0.22, 0.27)	0.05 (− 0.20, 0.31)
52 weeks	1.01 (0.16)	0.96 (0.23)	1.05 (0.25)	−0.05 (− 0.21, 0.12)	−0.05 (− 0.27, 0.18)	−0.002 (− 0.22, 0.22)
EuroQoL, /100						
0 weeks	82.7 (12.9)	83.1 (11.2)	80.4 (16.9)	–	–	–
6 weeks	80.6 (11.8)	83.0 (11.6)	83.9 (13.4)	1.6 (−5.5, 8.6)	4.9 (−4.2, 14.0)	3.3 (−5.3, 11.9)
12 weeks	83.1 (9.9)	86.2 (8.9)	85.9 (13.6)	2.4 (−4.1, 8.8)	4.9 (−3.8, 13.6)	2.5 (−5.7, 10.8)
26 weeks	86.3 (12.1)	87.8 (8.9)	87.2 (12.7)	0.9 (−6.5, 8.4)	2.6 (−7.1, 12.3)	1.7 (−7.1, 10.5)
52 weeks	88.5 (9.3)	86.9 (11.3)	85.3 (17.3)	−2.4 (−9.1, 4.3)	−1.2 (−11.6, 9.3)	1.2 (−8.7, 11.2)
Success, number of events/total sample size (percentage)					RR (95% CI)^‡^	
6 weeks	4/22 (18.2%)	8/26 (30.8%)	10/26 (38.5%)	1.69 (0.59, 4.87)	2.12 (0.77, 5.81)	1.25 (0.59, 2.66)
12 weeks	13/28 (46.4%)	19/35 (54.3%)	19/33 (57.6%)	1.17 (0.71, 1.93)	1.24 (0.76, 2.03)	1.06 (0.70, 1.62)
26 weeks	26/36 (72.2%)	27/35 (77.1%)	25/34 (73.5%)	1.07 (0.81, 1.40)	1.02 (0.77, 1.36)	0.95 (0.73, 1.25)
52 weeks	32/35 (91.4%)	31/33 (93.9%)	28/34 (82.4%)	1.03 (0.90, 1.17)	0.90 (0.75, 1.09)	0.88 (0.73, 1.05)
Recurrence, number of events/total sample size (percentage)					RR (95% CI)^∆^	
12 weeks	4/26 (15.4%)	8/30 (26.7%)	6/30 (20.0%)	1.73 (0.59, 5.10)	1.30 (0.41, 4.11)	0.75 (0.30, 1.90)
26 weeks	5/32 (15.6%)	9/35 (25.7%)	6/34 (17.6%)	1.65 (0.62, 4.40)	1.13 (0.38, 3.34)	0.69 (0.27, 1.72)
52 weeks	5/34 (14.7%)	10/32 (31.3%)	8/34 (23.5%)	2.13 (0.82, 5.54)	1.60 (0.58, 4.40)	0.75 (0.34, 1.67)

Combined = prolotherapy+physiotherapy; Success = completely recovered or much improved on the global rating of change scale; PFG = pain-free grip; PRTEE = Patient-Rated Tennis Elbow Evaluation; RR = relative risk; CI = confidence interval; SD = standard deviation

* *p* < 0.05; ^†^ between-group comparisons data from GEE analyses with positive results in favour of the first group; ^‡^ RR > 1.0 favours the first group; ^∆^ RR < 1.0 favours the first group

In terms of secondary outcomes, there were no significant differences between groups over time for any of the outcomes. However, self-reported worst pain (*p* < 0.001), PFG (*p* < 0.001) and EuroQol (*p* = 0.002) significantly improved over time in all three groups. In general, the Combined treatment led to more pain compared to other treatments. For reported worst pain, mean improvement was significant in Physiotherapy versus Combined treatments at all time points (e.g., mean improvement at 12 weeks = 1.74; 95% CI: 0.55 to 2.93; *p* = 0.004) or versus Prolotherapy within 12 weeks from baseline (e.g., mean improvement at 12 weeks = 1.37; 95% CI: 0.17 to 2.56; *p* = 0.03) (Table 2).

There were no significant differences between groups in the use of not-per-protocol treatments (*p* = 0.999), or frequency of use of any pain medication (None, 1–3 days/week, 4–7 days/week; *p* = 0.847) over the course of the trial. There were no significant adverse events in the Physiotherapy group. In the Prolotherapy group, one participant developed neuropraxia of the posterior interosseous nerve after the 4th treatment. This resolved over 3 months. Another participant developed painful bruising throughout the forearm after the 2nd treatment, which settled over 2 weeks.

Overall, the recurrence in elbow symptoms for all those assessed at 52 weeks was 23% with no significant difference in the recurrence rate between groups at any time (12, 26, 52 weeks: *p* = 0.98; Table 2).

### Predictors of response

A prespecified subgroup analysis of the success rates at 12 weeks follow-up was performed to look for any predictors of response amongst demographic or clinical variables. No difference in success rates was found, with and without adjustment for treatment groups, side affected, type of work (manual/non-manual/not working), perceived cause (extrinsic/intrinsic), or recurrence at 26 or 52 weeks. At 6- and 12-weeks follow up, adequate compliance with the exercise program was reported by 68 and 40% of the Physiotherapy group, and 63 and 48% of the Combined group, respectively. There were no significant differences in the proportions of success at 12 weeks or the risk of relapse at 52 weeks when comparing adequate with inadequate compliance.

### Economic analysis

In Australian dollars, a course of prolotherapy treatment was estimated to cost $495 for both unilateral cases and $631 for bilateral cases. A course of physiotherapy cost $415 and $475 for unilateral and bilateral cases respectively. Combined treatment cost $815 and $1011 per course for unilateral and bilateral cases, respectively. The average non-protocol treatment costs in the Prolotherapy group were $3, compared with $59 in the Physiotherapy group and $6 for the Combined group. This difference was not statistically significant and was mostly due to large costs incurred by a single trial participant in the Physiotherapy group.

## Discussion

This RCT, comparing dextrose prolotherapy, a physiotherapy program of manual therapy and exercise, and the two combined for adults with LE, found no difference between groups for the primary outcomes at short- and long-term follow-up. There were consistent and significant improvements in PRTEE scores from baseline at each follow-up time point for all groups. Without a placebo/control group, the attributable effect for each treatment over natural history is unknown. However, it can be estimated by comparison with other studies comparing these treatments with placebo or minimal intervention groups.

For prolotherapy, outcomes in the current study are consistent with a pilot level study in participants with LE whose baseline characteristics were of slightly greater severity and duration [7]. Using a similar intervention delivered under ultrasound guidance with an identical injectant, the PRTEE outcomes for prolotherapy were significantly better than for a control group at 8 and 16 weeks, and were less than the improvements in PRTEE seen in the current study at 6, 12 and 26 weeks. Our findings are also consistent with a second pilot level study which compared prolotherapy to normal saline [8], though direct comparison is again limited because the participants in that pilot study had more severe symptoms at baseline and the prolotherapy solution contained sodium morrhuate in addition to dextrose, which may have a different mechanism of action [8].

In terms of physiotherapy, two previous RCTs [4, 5] have investigated the effects of a physiotherapy intervention identical to the one used in this current study, except for the dose (8 treatment sessions in the previous studies versus 4 treatment sessions in the current study). While the baseline demographic and clinical characteristics were similar between all three studies, the number of participants in the physiotherapy groups who achieved a successful outcome at 12 weeks were significantly greater in the previous studies (Bisset et al. 65%, Coombes et al. 73%) compared to our success rate (47%), with a RR of 2.3 (95% CI 1.3 to 4.1) [4] and 2.0 (95% CI 1.1 to 3.5) [5], respectively. This suggests that the number of treatment sessions may substantially influence treatment effects for physiotherapy, with a greater treatment effect likely with an increased number of treatment sessions. Further meta-analysis or pooling of individual patient data across studies may be warranted, in order to further explore the dosage effects associated with physiotherapy treatment.

The pattern of recovery identified in the current study suggests that physiotherapy may offer more rapid improvement over prolotherapy. Alternatively, the more rapid improvement seen in the physiotherapy group may reflect the more compressed treatment schedule (i.e., weekly treatment sessions in physiotherapy compared to 4-weekly sessions in prolotherapy). Importantly, the combined treatment did not improve outcomes beyond that of prolotherapy or physiotherapy alone. This is the first study to compare dextrose prolotherapy to physiotherapy, and the first to compare either one to a combined therapy.

The results of this study are strengthened by the robust study design, including successful blinding of the outcome assessor, concealed allocation of participants, the minimal number of drop outs, and the intention-to-treat analyses. The main limitation is the lack of a control or placebo group, particularly as previous studies have reported up to 93% success at 52 weeks’ follow-up for participants in a wait-and-see or placebo injection group [5, 6]. Using control/placebo group data extracted from previous RCTs with similar populations [5, 6], meta-analyses of success rates revealed no significant difference between placebo injection and prolotherapy in the short term (4 to 6 weeks: RR 1.86, 95%CI 0.52 to 6.74), but a significant difference between placebo injection and physiotherapy (RR 3.94, 95%CI 1.38 to 11.27) [5, 6]. Similar findings occurred at 12 weeks’ follow-up: RR (95% CI) 1.59 (0.85 to 2.95) and 1.97 (1.13 to 3.44) for prolotherapy and physiotherapy, respectively, compared to placebo injection [5, 6]. These findings are consistent with previous work [5, 6] that found that physiotherapy was superior to placebo/control in the short-term.

Another limitation is that 18% of the total study participants had a symptom duration of < 12 weeks at enrolment. This subgroup of participants with a shorter duration of elbow pain may exhibit a more favourable natural history response, or may respond differently to the different treatments in this study. In clinical practice, prolotherapy injections are often a treatment of choice for more chronic conditions when more conservative treatments have failed [25]. Individuals with recalcitrant LE may benefit more from prolotherapy compared to other treatments. Nonetheless, a post hoc subgroup analysis excluding participants with symptoms for < 12 weeks showed the same conclusions of no significant Group effect for PRTEE (*p* = 0.24), with all three groups showing a significant improvement in PRTEE over time (*p* < 0.001). Future research should consider stratifying individuals based on the duration and severity of condition at baseline.

The results may have been biased by differential use of medications and non-protocol treatments between groups. However, such treatments were used by a small minority in all groups and adjustment for differences between groups made no difference to response rates and conclusions.

Our study was limited in its power to detect small differences between treatments and in the predictors of response. Our sample size of 40 per group was calculated on detecting a minimum clinically important difference in PRTEE outcomes of 13/100, a conservative difference based on a past trial of LE [11]. This setting ensures that any significant differences between groups identified are of clinical importance. Indeed, relative performance of treatments was assessed and reported in terms of the relative effect sizes, not by the statistically significance alone.

The mechanism of prolotherapy in overuse tendinopathy such as LE is unclear and likely multifactorial. Hypertonic dextrose is hypothesised to stimulate healing of chronically injured connective tissue [9]. In vivo studies have reported increased inflammatory markers [26] and significantly enlarged cross-sectional area in animal models with medial collateral ligament [27] and carpal tunnel pathology [28]. In addition to dextrose-specific effects, needle trauma and tissue-specific volume effects have also been documented [29]. Prolotherapy mechanisms may also include stimulation of growth factor release favouring soft tissue healing [30–32]. A recent systematic review and RCT found that structural characteristics in tendons including lateral elbow tendinopathy, are not correlated with clinical severity, and that other mechanisms may explain changes in pain and function associated with tendinopathy [33, 34]. Glucose injections may have a pain-specific neural effect as was suggested in an RCT showing significant and sustained benefit of perineural injections with 5% glucose over saline injections in carpal tunnel syndrome [35]. Given that sensorimotor changes have been consistently demonstrated in LE [36, 37], research into perineural injections of the radial nerve and its branches may be worth pursuing. This study, while not definitive, offers new information about the comparative effectiveness of physiotherapy and prolotherapy in a cohort of patients with predominantly chronic LE; each may be equally effective for LE, but it is not clear how much they influence the natural history of the condition. Using both treatments together seems no better than each used alone. For physiotherapy, a compressed treatment schedule may hasten recovery and other research suggests more treatment sessions may increase early success rates. The role of prolotherapy for patients with more chronic LE refractory to other treatments, reflective of its usual application in clinical practice, warrants further investigation.

## Conclusion

Four sessions of prolotherapy injections or four sessions of physiotherapy both improved pain and function over 52 weeks in people with LE, with no significant difference between groups. Combining these treatments did not further improve outcomes in LE, so single modality treatments (prolotherapy or physiotherapy) are recommended to minimise cost.

## Data Availability

Data and material related to this study is available from the corresponding author on request.

## Abbreviations

ANOVA: Analysis of variance
CI: Confidence interval
GEE: General estimating equation
GIC: Global impression of change
LE: Lateral epicondylalgia
MCAR: Missing completely at random
MWM: Mobilisation-with-Movement
PFG: Pain-free grip
PPT: Pressure pain threshold
PRTEE: Patient-Rated Tennis Elbow Evaluation
RCT: Randomised control trial
RR: Relative risk
SD: Standard deviation
USA: United States of America
